# Use of a Combination Strategy to Improve Morphological and Functional Recovery in Rats With Chronic Spinal Cord Injury

**DOI:** 10.3389/fneur.2020.00189

**Published:** 2020-04-02

**Authors:** Roxana Rodríguez-Barrera, Adrián Flores-Romero, Vinnitsa Buzoianu-Anguiano, Elisa Garcia, Karla Soria-Zavala, Diego Incontri-Abraham, Marcela Garibay-López, Juan José Juárez-Vignon Whaley, Antonio Ibarra

**Affiliations:** ^1^Centro de Investigación en Ciencias de la Salud (CICSA), Facultad de Ciencias de la Salud, Universidad Anáhuac México Campus Norte, Huixquilucan, Mexico; ^2^Proyecto CAMINA A.C., Mexico City, Mexico; ^3^UIMEN, CMN Siglo XXI, Mexico City, Mexico

**Keywords:** SCI, Immunomodulation, MSC, Fibrin tissue adhesive, A91 peptide

## Abstract

Immunization with neural derived peptides (INDP), as well as scar removal (SR) and the use of matrices with bone marrow-mesenchymal stem cells (MSCs), have been studied separately and proven to induce a functional and morphological improvement after spinal cord injury (SCI). Herein, we evaluated the therapeutic effects of INDP combined with SR and a fibrin glue matrix (FGM) with MSCs (FGM-MSCs), on motor recovery, axonal regeneration-associated molecules and cytokine expression, axonal regeneration (catecholaminergic and serotonergic fibers), and the induction of neurogenesis after a chronic SCI. For this purpose, female adult *Sprague-Dawley* rats were subjected to SCI, 60 days after lesion, rats were randomly distributed in four groups: (1) Rats immunized with complete Freund's adjuvant + PBS (vehicle; PBS-I); (2) Rats with SR+ FGM-MSCs; (3) Rats with SR+ INDP + FGM-MSCs; (4) Rats only with INDP. Afterwards, we evaluated motor recovery using the BBB locomotor test. Sixty days after the therapy, protein expression of TNFα, IL-4, IL-10, BDNF, and GAP-43 were evaluated using ELISA assay. The number of catecholaminergic and serotonergic fibers were also determined. Neurogenesis was evaluated through immunofluorescence. The results show that treatment with INDP alone significantly increased motor recovery, anti-inflammatory cytokines, regeneration-associated molecules, axonal regeneration, and neurogenesis when compared to the rest of the groups. Our findings suggest that the combination therapy (SR + INDP + FGM-MSCs) modifies the non-permissive microenvironment post SCI, but it is not capable of inducing an appropriate axonal regeneration or neurogenesis when compared to the treatment with INDP alone.

## Introduction

Spinal cord injury (SCI) leads to a series of anatomical and physiological changes resulting in permanent or temporary changes in spinal cord (SC) functionality (1, 2). Cell necrosis, glial reaction and especially inflammation induces the appearance of cavities, cysts and glial scars which interrupt the descending and ascending axonal tracts causing paraplegia or quadriplegia (3).

Infiltration of inflammatory cells has been correlated with the amount of tissue damaged after injury. However, it has been shown that the presence of inflammatory cells is essential for neuroprotection (4, 5) and regeneration (6, 7) of the SC, where its action depends on the effects exerted by autorreactive Central Nervous System (CNS) T-cells which are part of an autoimmune response developed after injury (4).

Protective autoimmunity (PA) is a new concept, which refers to the modulation of self-reactive mechanisms to promote neuroprotection and neural restoration (3, 4). PA is a physiological mechanism induced after SCI that could be boosted by immunizing with neural-derived peptides (INDP), a therapeutic strategy that requires the use of peptides such as A91 to induce PA. A91 is a peptide derived from the immunogenic sequence (87–99) of the myelin basic protein (MBP), the most immunogenic protein in the CNS. Activating T-cells by means of A91_87−99_ peptide, induces a Th2 response, which stimulates microglia to its differentiation to an M2 phenotype, resulting in a micro-environment with lower production of free radicals, improved motor recovery, among other neuroprotective mechanisms (5, 8, 9) as well as axonal regeneration (7, 10). This therapeutic strategy has rendered encouraging results both, in acute and chronic SCI, being the chronic one the most difficult stage of injury to carry out a therapeutic approach.

In the chronic phase of SCI there is scar tissue avoiding a correct reconnection of axons by forming a physical and chemical barrier made of sulfated proteoglucans and CD44 glycoprotein, thus inhibiting the formation of growth cones and axonal prolongation (11, 12). Additionally, chronic SC injury is considered a period of low activity with a progressive decline. Therefore, in order to achieve axonal regeneration a possible alternative could be scar removal (SR), with the goal of re-establishing electrical conduction and consequently the synapses, in addition to restoring the conditions of an acute injury, such as activation of PA as wells as cytokine release and neurotrophic factors (7, 13). On the other hand, biomaterials have been used such as scaffolds to facilitate regeneration of nerve fibers (12). Fibrin glue matrix (FGM) is a fibrinogen and thrombin derivative that has been used as a cavity repair adhesive material (14). This biomaterial has successfully been used in combination with bone marrow -mesenchymal stem cells (MSCs) to promote the secretion of neurotrophic factors, giving the opportunity for a neurogenic effect and axonal regeneration (10, 15). FGM is a biocompatible element with MSCs (16). As PA has proven to exert beneficial actions after a chronic SCI, we now intended to improve this effect by combining INDP therapy with other therapeutic strategies that also have shown beneficial effects and that could complement the actions provided by INDP. The present study evaluates the effect of combining INDP plus surgical removal and inhibition of glial scar, together with a FGM impregnated with MSCs. We expect that the renewed microenvironment induced by SR and, its inhibition, will allow INDP to promote neuroprotection (after SR) and induce the production of neurotrophic factors and thus axonal regeneration and neurogenesis (7, 11, 17). In the same way, MSCs could modulate SR (18), reduce proinflammatory cytokines (19) and collaborate to induce axonal regeneration (20). Finally, FGM will provide the matrix for MSCs and the scaffold for axonal growing (21). It is plausible to expect that, the combination of these strategies, could integrate different beneficial elements that may result in a better neurological recovery, especially in the chronic stage of injury (a hostile phase for regeneration).

A recent study in our research group proved that the combined therapy of SR + INDP + FGM-MSCs in a model of complete transection improved motor and electrophysiological recovery with an increase in genes associated with regeneration and an increment in the percentage of serotonergic fibers (5-HT) (10).

In this study, we evaluated the effect of this combination therapy on motor recovery, protein expression of cytokines and molecules associated with regeneration, axonal regeneration and neurogenesis in a model of chronic contusion of SCI.

## Materials and Methods

### Experimental Design

Sample size for this experiment was calculated using an alpha of 0.05 and beta of 0.20. Experiments were performed 60 days after SCI, with subsequent analyses carried out over the two following months. The experiment consisted of 48 rats with chronic SCI randomly distributed into four groups (GraphPad QuickCalcs: http://www.graphpad.com/quickcalcs/): (1) rats immunized with complete Freuds adjuvant + PBS (control; PBS-I) (n = 12); (2) rats only with INDP (n = 12); (3); rats with SR + FGM-MSCs (n = 12) and (4) rats with SR + INDP + FGM-MSCs (n = 12).

Locomotor function was evaluated 60 days after SCI and thereafter weekly throughout 2 months. At the end of each experiment (120 days after SCI), rats were euthanized, and the SC was then analyzed. We determined the neurogenic effect of the therapy in all studied groups at the injured site of the SC, as well as the expression of proteins for brain-derived neurotrophic factor (BDNF), growth associated protein-43 (GAP-43), interleukin-4 (IL-4), interleukin-10 (IL-10), and tumor necrosis factor alpha (TNFα). We determined the number of 5-HT+ and TH+ fibers in the caudal stump of the four groups. Finally, neurogenesis by Immunofluorescence was also assessed.

### Ethics Statement

All procedures were carried out in accordance with the National Institutes of Health Guide for the care and use of laboratory animals, and the Mexican Official Norm on Principles of Laboratory Animal Care (NOM 062-ZOO-1999). In addition, the Animal Bioethics and Welfare Committee approved all animal procedures (ID: 178544; CSNBTBIBAJ 090812960). All experiments were designed and reported according to the ARRIVE guidelines.

In order to perform euthanasia, animals were previously anesthetized by an intramuscular injection of a mixture of ketamine (50 mg/kg) and xylazine (10 mg/kg).

### Spinal Cord Injury

Adult female Sprague–Dawley rats (13–14 weeks old) weighing between 230 and 250 g were subjected to a moderate SC contusion. Animals were anesthetized by an intramuscular injection of a mixture of ketamine (77.5 mg/kg) (Probiomed, Mexico City, Mexico) and xylazine (12.5 mg/kg) (Fort Dodge Laboratories, Fort Dodge, Iowa, USA). One hour after induction of anesthesia, their skin was opened in layers and a laminectomy was performed at T9 vertebral level of the SC. Subsequently, a 10 g rod was dropped onto the SC from a height of 25 mm using the NYU impactor (NYU, New York, USA). Functional recovery of all groups was assessed using the BBB locomotor scale.

### Postoperative Care

After SCI, animals were housed with food and water ad libitum, and received manual bladder voiding, three times a day for 2 weeks. Sterile bedding and filtered water were replaced daily. To avoid infection, Enrofloxacin (Marvel, Mexico City, Mexico) was diluted into their drinking water at an approximate dose of 64 mg/kg/day for 1 week. Animals were carefully monitored for signs of infection, dehydration, or auto mutilation with appropriate veterinary assistance as needed.

### Antigen (A91_87−99_ Peptide)

A91_87−99_ peptide was derived from the encephalitogenic amino acid sequence 87–99 of the MBP. A non-encephalitogenic analog was obtained by replacing the lysine residue with alanine at position 91. The modified peptide was purchased from Invitrogen Life Technologies (San Diego, CA, USA). Reverse-Phase HPLC confirmed the purity of the A91_87−99_ peptide (>95%).

### Isolation and Phenotyping of MSCs

MSCs were obtained from female Sprague Dawley healthy rats. Animals were euthanized with an overdose of pentobarbital sodium. Bone marrow was obtained from both femurs and tibias using a 200 μL micropipette and deposited in a 15 mL conical tube with culture medium [Dulbecco's Modified Eagle [DMEM], from GIBCO]. The sample was centrifuged at 1500 rpm for 7 min. Cells were then separated using a Ficoll (Sigma-Aldrich) (3 mL) gradient centrifuged at 2,000 rpm at 24°C for 30 min. The total number of nucleated cells obtained was quantified and 9 × 10^6^ cells were seeded into a 75 cm^2^ culture flask (in 5 mL of DMEM with 20% fetal bovine serum (FBS), from GIBCO), 1 mL of L-Glutamine (GIBCO), 5 mL of HEPES (Sigma-Aldrich), and 1 mL of Penicillin-Streptomycin (GIBCO). Cells were then placed in a water-jacketed incubator at 37°C with 5% CO2 until they formed a fibroblast monolayer. Finally, MSCs were reseeded onto the fibroblast layer and maintained for 2 weeks until transplantation.

#### Phenotyping

MSCs expanded in culture from passage 4 were phenotypically characterized by flow cytometry. The cells were centrifuged at 1,500 rpm for 7 min and they were blocked for 30 min with normal serum, then the cells were centrifuged at 1,500 rpm for 7 min to wash. Afterwards the cells were incubated with primary antibodies Anti rat- CD13 Santa Cruz Biotechnology Inc; CD90.1 eflour 450 anti-rat (Biolegend); CD70 PE anti-rat (Biolegend); PE/Cy7 anti-mouse CD105 Biolegend; Anti rat-CD117 Millipore; CD34 (ICO 115) FITC (Santa Cruz Biotechnology Inc), all at a dilution of 1:1000 in darkness for 30 min at 4°C. They were washed twice with FACS Buffer before being centrifuged again at 1,500 rpm for 5 min finally being quantified and analyzed with flow cytometry using the Cell Quest-Pro (BD Bioscience) program.

Cell phenotype proportion: 99% of the cells were mature MSCs; 80.45% were positive for CD13 (marker for subpopulation of MSCs); 51% were double positive for CD105+CD90+ (marker for subpopulation of MSCs); 28.1% were positive for CD70 (marker for subpopulation of MSCs); 11.49% were positive for CD 117 (marker for subpopulation of MSCs) and 1.70% of cellular population was positive for CD34 (specific control marker for hematopoietic stem cells) (10). Therefore, the majority of cells transplanted were adult MSCs.

### Intervention in the Chronic SCI (Combination Therapy)

Sixty days after SCI, animals were anesthetized again as previously described. Then after, using a surgical microscope, a longitudinal incision was performed and fibrosis was removed. A second longitudinal incision was carried out, the meninges were referenced to the bordering muscles and the necrotic tissue was eliminated. The scar from each stump was removed by performing a single incision with a double-bladed scalpel leaving a space of 2 mm in length. Surgery for this procedure is a reproducible technique. The surgeon was always blinded to the group of animals. This method is helpful to successfully remove the glial scar and it does not generate any additional neurological deficit. Importantly, it produces a mild lesion that allows a renewed production of growth factors and the consequent induction of a favorable microenvironment for neural regeneration. Once the scar was removed, its renewal was halted by adding α,α′- dipyridyl (DPY), an iron chelator that inhibits a key enzyme for collagen biosynthesis in the acute phase of SCI. Therefore, this iron chelator inhibits SR and promotes an extensive long-distance regeneration of injured axons. DPY was injected directly –six times– into each stump of the SC by using a Hamilton syringe. Each injection deposited 2 μL volume of DPY (16 nmol) diluted in PBS. Right after, a mixture of MSCs were pre-labeled with the membrane dye PKH26, (Sigma-Aldrich) then 2.5 × 10^6^ MSCs in 5 μL and FGM (10 μL, with a final concentration of fibrinogen of 5 mg/mL) was grafted using a Hamilton syringe. Finally, the meninges were sutured with a 9-0 suture and the aponeurotic plane and skin were separately sutured with a nylon thread. Rats were then immunized subcutaneously at the base of the tail with 200 μg of A91_87−99_ peptide dissolved in phosphate buffered saline (PBS), emulsified in an equal volume of complete Freund's adjuvant (CFA) containing 0.5 mg/ml Mycobacterium tuberculosis (Sigma-Aldrich, St. Louis, MO, USA). In order to boost the protective and regenerative action of INDP, immunization with A91_87−99_ peptide was accompanied with CFA, an adjuvant that potentiates the immune response but does not influence the protection or restoration exerted by INDP (7, 10).

### Assessment of Motor Recovery

Locomotor recovery was assessed using the Basso, Beattie & Bresnahan (BBB) open-field locomotor scale method. Animals were evaluated 60 days after SCI and thereafter weekly throughout 8 weeks. Three separate blinded observers evaluated all animals and the average of the three scores was used.

### Enzyme-Linked Immunosorbent Assay (ELISA)

Two months after therapeutic intervention, animals were euthanized with an overdose of pentobarbital sodium (80 mg/kg) and then, SC samples were rapidly excised. Reagents, samples, and standards were prepared according to the instructions provided by the manufacturer: IL-4 ELISA Kit (Cell Applications, San Diego, CA, USA), IL-10 ELISA kit (RayBiotech, Norcross, GA, USA), BDNF ELISA Kit (Ray Biotech, Norcross, GA, USA), neuromodulin (GAP-43) ELISA kit (CUSABIO, Houston, TX, USA) and TNFα ELISA kit (Origene, Rockville, MD, USA). Briefly, 100 μl standard or 30 μg total protein sample were added to each well and incubated for 2 h at 37°C. The liquid of each well was removed and not washed. Afterwards, 100 μl of Biotin-antibody (1x) was added to each well and incubated for 1 h at 37°C, followed by aspiration and washing for 3 times. One hundred μl HRP-avidin (1x) were added to each well and incubated for 1 h at 37°C. Subsequently, it was aspirated and washed 5 times and, 90 μl of TMB substrate were added to each well, incubated and protected from light for 15–30 min at 37°C. Finally, 50 μl Stop solution were added to each well and read at 450 nm within 5 min.

### Immunohistochemistry for 5-HT+ and TH+ Fibers

Two months after the therapeutic intervention, animals were euthanized with an overdose of pentobarbital sodium (80 mg/kg) and an intracardiac perfusion with 4% paraformaldehyde was performed. The affected portions of the SC were fixed overnight and then transferred to 30% sucrose for cryoprotection. Samples were embedded in Tissue-Tek (Miles Elkhart, IN, USA), and longitudinal frozen sections (40 μm thick) were performed. Immunohistochemical staining was carried out in order to count the amount of TH+ and 5-TH+ fibers. Tissues were incubated in 0.03% hydrogen peroxide to quench endogenous peroxidase activity. Subsequently, the tissue was incubated overnight with the following primary antibodies: monoclonal goat antibody against TH (1:2000; Chemicon), or polyclonal rabbit antibody against 5-HT (1:2000; Sigma- Aldrich). Following rinsing with PBS, samples were incubated for at least 2 h with donkey IgG anti-goat IgG (1:500; Chemicon) and Sheep IgG anti rabbit IgG (1:500; Abcam) secondary biotinylated antibodies. To visualize positive fibers, samples were incubated 5 min with Vector DAB kit (Vector laboratories, CA, USA). Then, samples were evaluated and analyzed by a blinded observer that counted individual fibers using a 20X objective (Olympus DP72, Japan). The number of regenerating axons 1 mm caudal to the lesion was assessed.

### Immunofluorescence for Evaluating Neurogenesis

Neurogenesis was evaluated by immunofluorescence using a double stain with anti-5-bromo-2'-deoxyuridine (BrdU) and doublecortin (Dcx) antibodies. BrdU is a synthetic nucleotide analog of thymidine incorporated during the S phase of the cell cycle, whereas Dcx is a marker for neural progenitor cells (NPCs). Therefore, BrdU+/Dcx+ cells are a result of neurogenesis. For this assay, the rats received one injection of BrdU (Abcam, Cambridge, UK; 50 mg/kg) intraperitoneally every 12 h for 5 days. The SC samples were then removed (1.0 cm caudal/rostral from the injury site) perfused and fixed with 4% paraformaldehyde. Tissues were cut transversally with the cryostat into sequential serial sections (at 0, 2, 4, and 6 mm caudal and rostral from the epicenter). Slices were 40 μm thick and a total of 48 sections per animal were counted and placed on slides using the free float method. Slides were washed twice for 10 min with PBS-Triton (PBT) and incubated with ImmunoRetriever (Bio SB, Santa Bárbara, CA, USA) for 60 min at 65°C. Afterwards, slides were washed three times for 5 min with PBS and incubated for 30 min with 1N HCl at 37°C. When completed, they were incubated for 10 min with sodium borate 0.1 M and washed three times with PBT. Unspecific binding sites were blocked with standard blocking solution with fetal bovine serum for 30 min. The primary antibodies against BrdU (Roche Diagnostics, Indianapolis, USA) (mouse IgG, 1:250) and Dcx (Santa Cruz Biotechnology, Dallas, TX, USA) (goat IgG, 1:250) were allowed to incubate for 20 h overnight. The next day, the slides were washed three times for 10 min with PBT and incubated with secondary antibodies (Invitrogen, Carlsbad, CA, USA) (BrdU: donkey IgG; Dcx: rabbit IgG; all at 1:500) for 2 h. The excess antibodies were removed by washing with PBT. Slides were counterstained with DAPI. All areas were quantified as total number of cells in all SC samples by a blinded evaluator using cell counting software ImageJ 1.52a. The total number of BrdU+/Dcx+ cells was obtained by averaging the total number of cells from 3 slides (3) and confocal images were acquired using a Zeiss LSM 800 microscope.

### Statistical Analysis

Data is displayed as mean ± standard deviation (SD), and statistical significance was established when *p* ≤ 0.05. GraphPad Prism 8.0 (GraphPad Software, Inc. La Jolla, CA, USA) was employed in statistical analysis. Data from the assessment of functional recovery was analyzed using an ANOVA for repeated measures with Bonferroni's *post hoc* test (BBB test). Protein expression, the percentage of 5-HT+ and TH+ fibers and neurogenesis were analyzed by One-way ANOVA followed by Tukey–Kramer *post hoc* test.

## Results

### INDP Alone Induces the Best Locomotor Recovery After Chronic SCI

**Figure 1:**
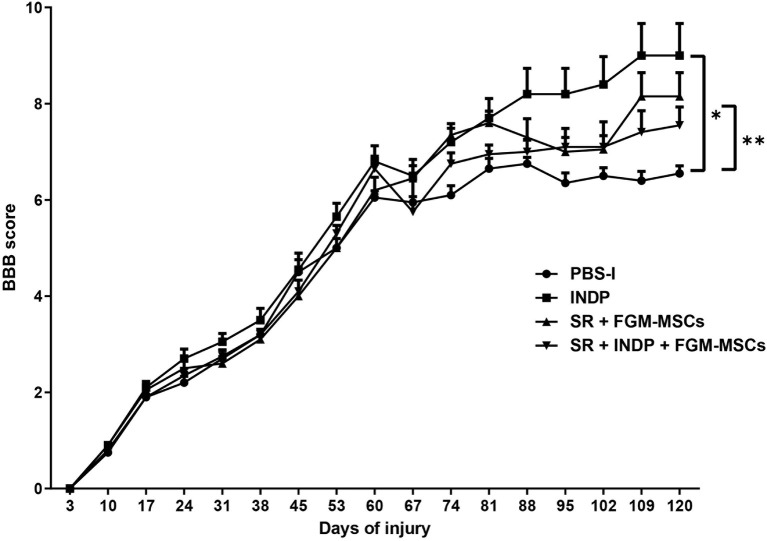
Motor recovery after therapeutic intervention. After treatment, a significantly better motor recovery was observed in SR+FGM-MSCs and INDP alone groups. Rats treated with INDP alone showed the highest motor recovery among all groups. *p = 0.0220, **p = 0.045, ANOVA for repeated measures with Bonferroni's *post hoc* test. Each point represents the mean ± SD of 12 rats.

In order to test the effect of the different therapeutic strategies on neurological recovery, the motor performance was evaluated comparing the four groups. Figure 1 shows that 60 days after injury the locomotor performance of all groups was very similar before the therapeutic intervention (6.45 ± 0.84, mean ± SD; p = 0.1020, One-way ANOVA followed by Tukey–Kramer *post hoc* test). Sixty days after the respective therapy, all treated groups presented an improvement in motor recovery when compared to the one observed in the PBS-I group. Rats submitted to SR + FGM-MSCs and the ones treated with only INDP showed the highest motor recovery compared to the rest of the groups (8.15 ± 1.56 and 9.0 ± 2.10 respectively; *p* < 0.05, ANOVA for repeated measures with Bonferroni's *post hoc* test). Rats treated with the combination strategy presented a lower motor recovery (7.55 ± 1.21) as compared to those treated with SR+FGM-MSCs or INDP alone; but the improvement was still significantly higher when compared to PBS-I rats (6.550 ± 0.49).

### INDP but Not the Combination Therapy Generates a Permissive Microenvironment Where Anti-inflammatory Cytokines and Regeneration-Associated Molecules Are Increased

It has been reported that INDP induces a permissive microenvironment for neural restoration in the chronic stages of injury (4, 5). Based on these findings, we explored the induction of this permissive microenvironment by analyzing the production of one pro-inflammatory (TNFα) and two anti-inflammatory cytokines (IL-4; IL-10). Additionally, the production of specific regeneration-associated proteins such as BDNF and GAP-43 were also assessed.

**Figure 2:**
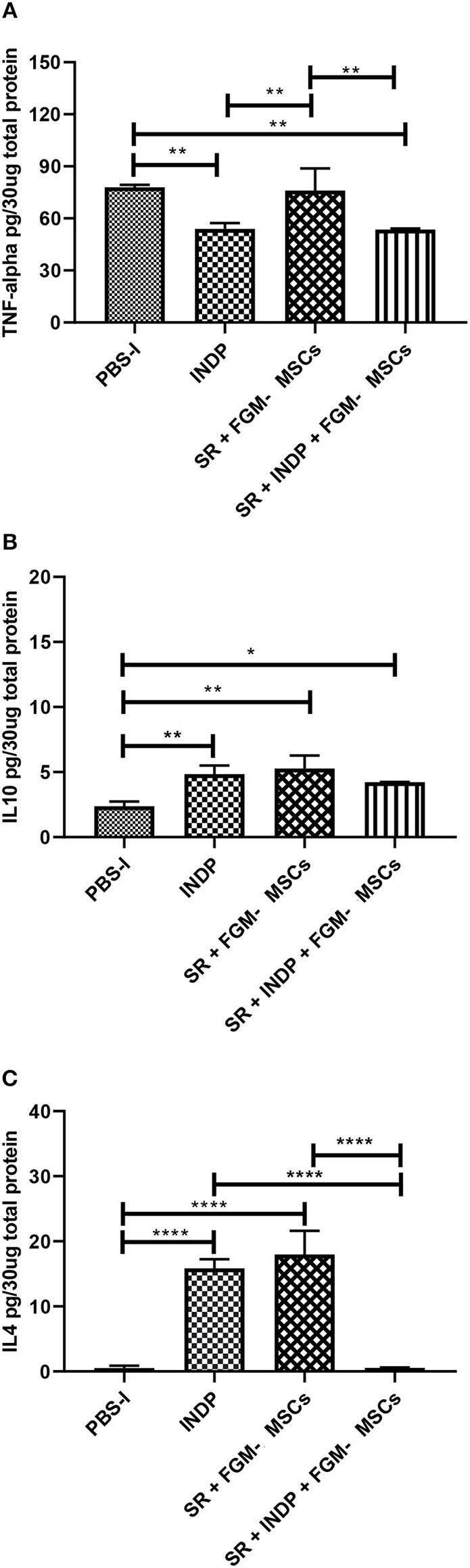
Cytokines concentration in the site of injury. TNFα **(A)** protein concentration was significantly reduced only in the INDP and SR + INDP + FGM-MSCs groups. Both IL-4 **(B)** and IL-10 **(C)** showed significantly increased levels of protein concentration in both INDP and SR + FGM-MSCs groups. *p < 0.05; **p < 0.01; ****p < 0.0001. One-way ANOVA followed by Tukey–Kramer *post hoc* analysis. Bars represent the mean ± SD of 4 rats. This is one representative graph of three experiments.

Figure 2A shows that INDP alone and SR + INDP + FGM-MSCs groups elicited a significant reduction of TNFα (53.91 ± 3.35 and 53.54 ± 0.66, respectively; mean ± SD) when compared to the rest of the groups (SR + FGM-MSCs: 76.02 ± 12.82 and PBS-I: 77.96 ± 1.33). When evaluating anti-inflammatory cytokines, a significant increase in IL-10 protein levels was observed in both INDP alone and SR + FGM-MSCs groups (Figure 2B; 4.84 ± 0.67 and 5.28 ± 0.99, *p* < 0.05, One-way ANOVA followed by Tukey–Kramer test) when compared to the SR + INDP + FGM-MSCs and PBS-I groups (4.22 ± 0.02 and 2.37 ± 0.36, respectively). Similarly, IL-4 protein levels significantly increased in the groups treated with INDP alone and SR + FGM-MSCs (15.85 ± 1.39 and 17.99 ± 3.63, respectively), when compared to the rest of the groups (Figure 2C; SR + INDP + FGM-MSCs: 0.56 ± 0.05 and PBS-I: 0.51 ± 0.36, *p* < 0.05, One-way ANOVA followed by Tukey–Kramer test).

**Figure 3:**
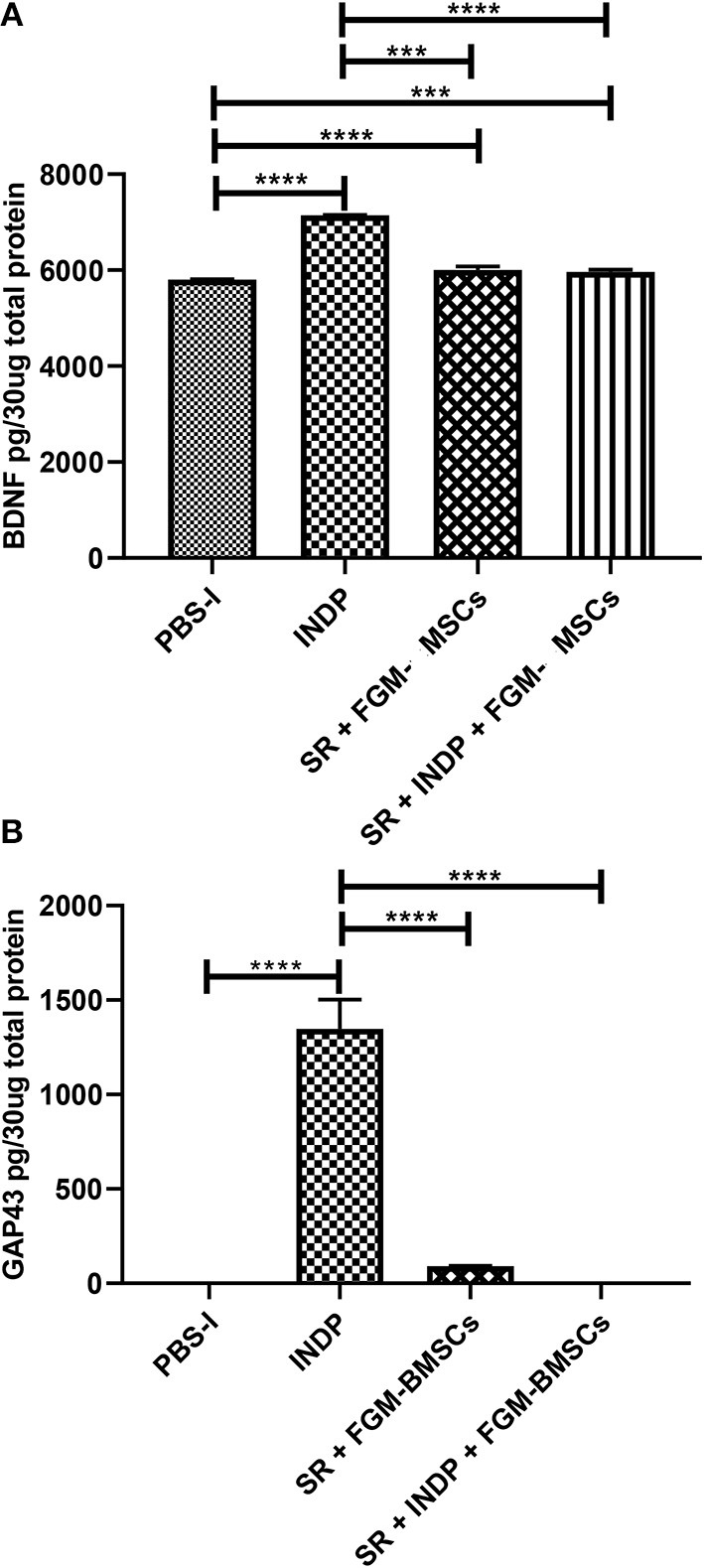
Regeneration-associated proteins concentration in the site of injury. BDNF **(A)** and GAP-43 **(B)** protein concentration was significantly increased only in the INDP group. ***p < 0.001;****p < 0.0001. One-way ANOVA followed by Tukey–Kramer *post hoc* analysis. Bars represent the mean ± SD of 4 rats. This is one representative graph of three experiments.

In regard to BDNF and GAP-43, which indicate regeneration at the site of injury, a significant increase in the protein concentration of these molecules was observed in the group treated with INDP alone (Figures 3A,B; BDNF:7144 ± 5.312; GAP-43:1347 ± 155.5). The rest of the groups presented lower values of BDNF (SR + INDP + FGM-MSCs: 5961 ± 46.57; SR + FGM-MSCs: 6006 ± 68.53; PBS-I: 5804 ± 3.87) and GAP-43 (SR + INDP + FGM-MSCs: 0.0001 ± 0.009; SR + FGM-MSCs: 91.52 ± 1.22; PBS-I: 0.13 ± 0.27).

### INDP Alone but Not the Combination Strategy Promotes Axonal Regeneration

To determine whether the microenvironment created by the different therapeutic strategies had any positive effects on axonal regeneration, we assessed the percentage of axons observed at the caudal stump of the SCI after therapeutic intervention. The percentage was obtained from the total number of fibers observed in sham-operated rats.

**Figure 4:**
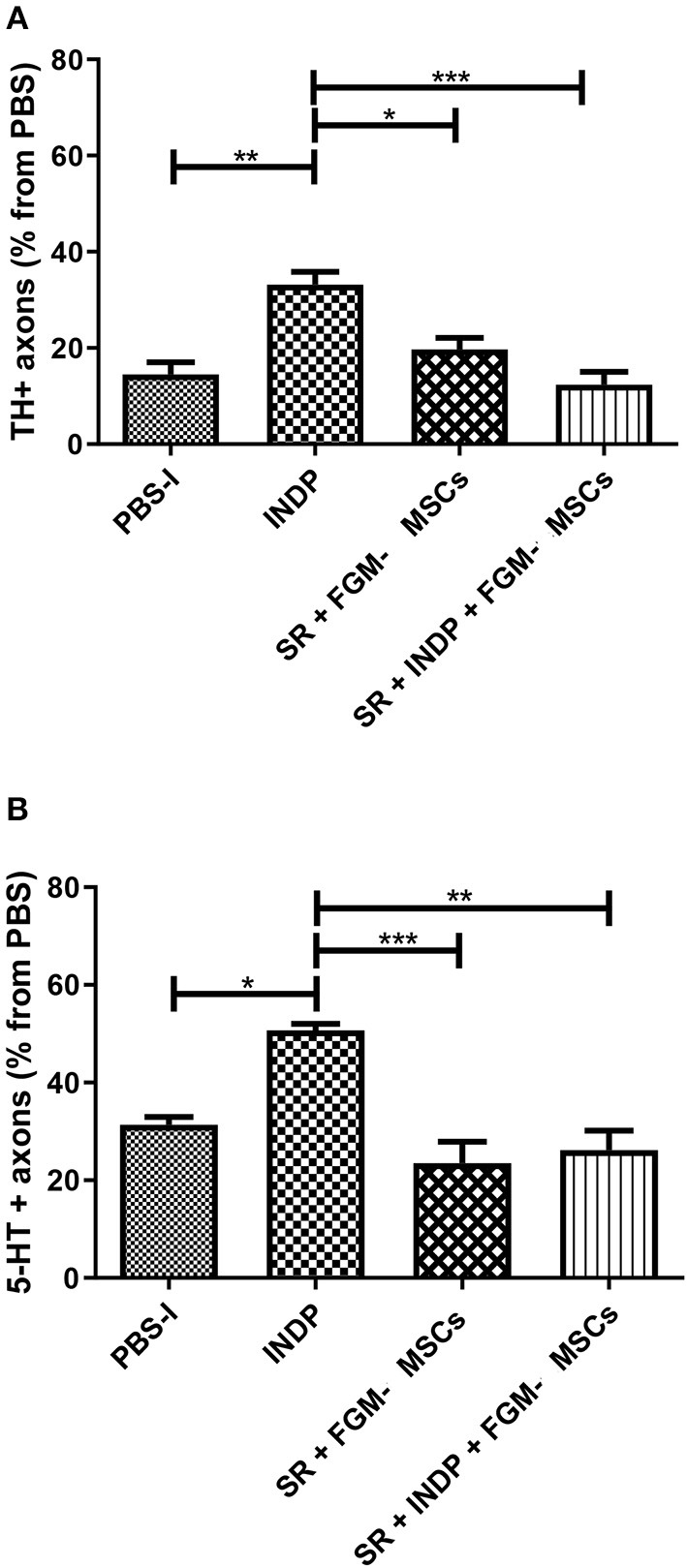
Percentage of axons observed at the caudal stump of SCI rats after therapeutic intervention. The percentage was obtained from the total number of axons found at the same level in sham-operated rats. INDP treatment induced a significant increase of both TH+ **(A)** and 5-HT+ **(B)** fibers (*p < 0.01; **p < 0.001; ***p < 0.0001, One-way ANOVA followed by Tukey–Kramer *post hoc* analysis). Animals treated either with SR + FGM-MSCs or SR + INDP + FGM-MSCs presented no significant differences in the percentage of TH+ or 5-HT+ fibers when compared to PBS-I rats. Bars represent the mean ± SD of 4 rats. This is one representative graph of three experiments.

**Figure 5:**
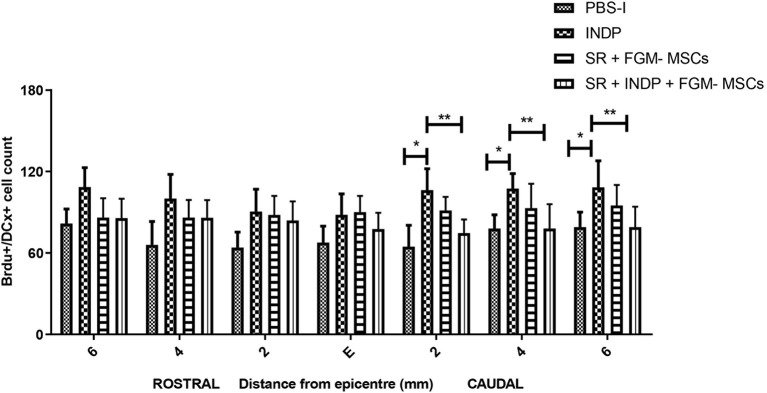
Number of BrdU+/DCX+ cells at caudal stumps of the SC. Rats treated with SR + FGM-MSCs or SR + INDP + FGM-MSCs showed no significant differences in the total number of BrdU/DCX labeled cells (neuroblasts) compared to the PBS-I group. Animals treated with INDP alone presented a significant increase in the total number of neuroblasts at the caudal stump of the SC. Bars represent the mean ± SD of 4 rats. This is one representative graph of three experiments. *p < 0.05; **p < 0.01; one-way ANOVA followed by Tukey's test.

Figure 4 shows the percentage of TH+ (Figure 4A) and 5-HT+ (Figure 4B) fibers in all groups. The group treated with INDP alone showed a significant increase in both TH+ and 5-HT+ fibers when compared to the rest of the groups (TH+ 33.17 ± 6.58; 5-HT: 50.67 ± 3.26; *p* < 0.05, One-way ANOVA followed by Tukey–Kramer test). Interestingly, groups treated with SR + FGM-MSCs or SR + INDP + FGM-MSCs presented no significant differences in the percentages of TH+ (SR + INDP+ FGM-MSCs: 12.33 ± 6.53; SR+FMG-MSCs: 19.67 ± 5.95) and 5-HT+ fibers (SR + INDP + FGM-MSCs: 26.17 ± 9.78; SR + FMG-MSCs: 23.50 ± 10.80) when compared to the PBS-I group (TH: 14.50 ± 6.18; 5-HT: 31.33 ± 3.93; *p* > 0.05).

### Neurogenesis Is Increased by INDP but Not by the Combination Therapy in Rats With Chronic SCI

**Figure 6:**
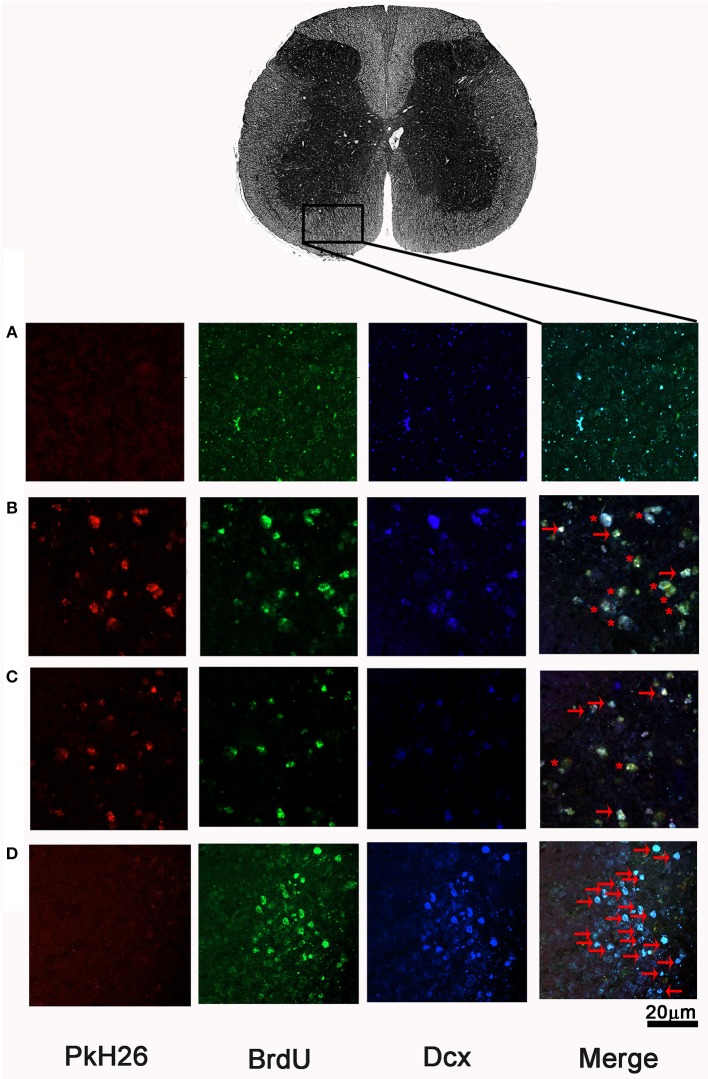
Representative microphotographs of BrdU+/DCX+ cells at ventral horns of SCI rats after therapeutic intervention. In the first section, pkH26 positive cells (red), BrdU+ cells (green), Dcx+ cells (blue). Double-label (BrdU+/Dcx+; cyan), triple-labeling (BrdU+/Dcx+/pkH26+; yellow) show merged final section. **(A)** PBS-I, **(B)** SR + FGM-MSCs, **(C)** SR + INDP + FGM-MSCs, and **(D)** INDP. An asterisk (*) indicates neuroblasts with triple labeling. Arrows depict BrdU+/DCX+ cells. A higher number of neuroblasts was observed in the group with INDP. This is one representative photograph of three experiments. Scale bar 20 μm.

In order to assess the number of NPCs at the injury site, we labeled BrdU+/DCX+ cells (neuroblasts) at the epicenter, rostral, and caudal stumps of the SC. Neither the rats treated with SR + FGM-MSCs nor SR + INDP + FGM-MSCs showed significant differences in the total number of neuroblasts when compared to the PBS-I group. However, the rats treated with INDP alone presented a significant increase in the total number of neuroblasts at 2, 4, and 6 mm caudal to the epicenter of the injury (Figures 5, 6); *p* < 0.05; One-way ANOVA followed by Tukey's test).

## Discussion

Previous studies in SCI models have shown that INDP is a promising therapeutic strategy to promote neuroprotection and neural restauration. These beneficial effects have been observed when INDP is administered in both acute and chronic phases of SCI (5, 10). Therefore, our purpose is to improve its protective and restorative benefits by combining INDP whit other successful therapeutic strategies. Previous investigations have shown that SR (22), the use of inhibitors of scar formation (17, 18, 22) or FGM-MSCs separately, promote a significant tissue restoration and motor recovery after SCI. Based on these results, combining INDP with these alternative strategies would be promising.

Chronic phase of SCI is a period of stability and low activity at the site of injury (11). In order to achieve a successful treatment, we proposed a surgical SR to break the latent chronic phase as this procedure reactivates the inflammation response (7) mimicking the initial injury process (19). This microenvironment, allows INDP to stimulate protective autoimmunity and thus, activate its neuroprotective (lipid peroxidation, inflammation and apoptosis inhibition) and restorative (IGF-1, BDNF, and GAP-43 induction) actions by modulating the gene expression of pro and anti-inflammatory cytokines as well as growth factors (7). Additionally, SR diminishes the existing physical barrier of collagen fibers and reactive glial cells. On the other hand, MSCs possess high immunomodulatory properties and they are capable of restoring injured CNS due to the anti-inflammatory molecules and trophic factors they produce to promote angiogenesis, remyelination, axonal regrowth, neural cell death inhibition, amongst others (20).

We predict that the conditions created by each of these therapies in the injury site and the synergy that they could generate together, should enhance the restoration conditions causing a better functional regeneration and thus, a better neurological recovery in the groups receiving the combined therapy. This therapeutic strategy has rendered optimistic results in SCI models involving acute contusion (5) or chronic complete transection (10). The objective of this study was to explore now the effects this combination could exert after a chronic SC contusion.

Our results show that INDP alone, SR + FGM-MSCs and the combination therapy (SR + INDP + FGM-MSCs) all increase motor recovery, but to different extents. Interestingly, the effect is reduced when the combination therapy is applied compared to the other treatment options. With the purpose of understanding this effect, the production of pro-inflammatory (TNFα) and anti-inflammatory cytokines (IL-4 and Il-10) were analyzed. We observed that there is a clear reduction of TNFα when INDP alone and the combination therapy were used, which prove how these therapies could modulate the inflammatory response, a phenomenon strongly involved in tissue destruction and restoration inhibition. On the other hand, we also found in both cases an increase in IL-10, an anti-inflammatory molecule that could help even more in the restoration process. Nevertheless, combination strategy did not promote the production of regeneration associated molecules neither improved the formation of regenerating fibers or neurogenesis. The lack of GAP-43 and BDNF—molecules favoring neural restoration- in the combination group could be -at least in part- the cause of the failure observed in these animals. It is difficult to understand why in this case, the microenvironment was not enriched with these molecules. SR for instance, has shown to improve the gene expression of BDNF and GAP-43 when combined with INDP. In the same way, this combination (SR + INDP) increased TH+ and 5-HT+ fibers and improved motor recovery (7). On the other hand, there is no evidence on any modulatory effect that could be exerted by FGM on the production of GAP-43 or BDNF. Therefore, the only option causing the lack of these molecules could be the counteraction induced by the modulating mechanisms exerted by MSCs. It is known that MSCs might inhibit the expansion of T cells not allowing the necessary response to promote the beneficial effects (21, 23). This is an issue that should be deeply studied in future investigations.

On the other hand, INDP alone was capable of providing a permissive microenvironment after a chronic SCI. This finding supports our previous reports and strengthens the idea that INDP is a promising therapy for acute and chronic SCI as now we show that INDP also promotes neurogenesis.

In the present study, INDP increased IL-10 concentrations. Regarding this, previous reports have stated that INDP alone produces an increase of this cytokine in chronic SCI (7) and stroke rat models (6). IL-10 is an anti-inflammatory cytokine that works together with CD4+ cells in glial maintenance (24) and as an apoptotic cascade blocker by increasing the levels of certain anti-apoptotic proteins such as Bcl-2 and Bcl-x leading to the decrease of Caspase-3 in motor neurons. This release of IL-10 in SCI models induces the recovery of motor function, decreases pain and tissue damage (25). In a recent study, IL-10 also proved to have NPCs homeostasis in the neurogenic niches by modulating the proliferative pathways of ERK and STAT 3 (26). Regarding IL-4, increased levels of this cytokine were found only in the group that received INDP alone and in the group treated with SR + FGM-MSCs. The fact that the combined therapy reduced IL-4 production is really interesting and could be, at least in part, the cause of its reduced morphological and functional effects. IL-4 can exert neuroprotective effects by regulating the acute and chronic response of the macrophages and promotes growth, phagocytic activity, and proliferation of microglial cells (27). IL-4 reduces also the production of nitric oxide and inflammatory cytokines such as, TNFα and INFγ (28–30). Another beneficial effect of IL-4 is neural restoration, this is induced by increasing the branching and maturation of oligodendrocytes through the interaction with the microglia (28). In addition, a study by Walsh et al. proves how this cytokine induces axonal growth in *ex vivo* models. Neurons incubated with IL-4 increased their axonal elongation as well as restoring injured neurons through the activation of neuronal receptors of IL-4 and so amplifying neurotrophin signaling via AKT and MAPK (31). Our current study indicates that INDP induces a favorable microenvironment of IL-4, thus suggesting its favorable actions at the injury site. This finding partially explains the motor recovery observed in this group, which could be due to the production of these cytokine, inducing maybe the production of neurotrophins such as BDNF (7, 32).

BDNF has an important function in neural tissue repair and CNS plasticity, especially in neurogenesis, axonal growth, myelination and sympathetic plasticity (33, 34). In fact, BDNF grants immediate actions together with direct effects on synaptic transmission (35). BDNF is also associated with GAP-43 induction, which is a common mediator of the regenerative effect of BDNF (36, 37). Notoriously, GAP-43 plays an essential role in the neurotrophic functions of BDNF (36) in cervical axotomy models where it was proven that the injection with BDNF stimulates expression of GAP-43, consequently inducing axogenesis and neural repair (38). These findings can explain the increase levels of GAP-43 observed in the group treated with INDP. GAP-43 is also strongly related in the transduction signaling for axonal growth and axons direction guidance (39). Several studies mention the possible role that GAP-43 has in the regulation of neurotransmitter release (36, 40). In summary, GAP-43 helps as a useful marker for neural regeneration and has an important role in neurites formation, regeneration and neuroplasticity (37).

All of these results, support the idea that BDNF together with GAP-43 contribute to neural restoration. However, to demonstrate whether the permissive anti-inflammatory microenvironment created by the combined therapy offered in our work had positive effects on axon regeneration, we evaluated the percentage of 5-HT and TH immunoreactivity fibers at the caudal portion where the lesion occurred prior to treatment. Axons which are positive to 5-HT and TH along the SC derive from regions near the brain stem and are distributed throughout the whole SC, these axon fibers descend from neurons located in the Raphe nucleus (41) and Locus Coeruleus (42), respectively. After SCI, the distal portions of the axons are isolated from the neural nuclei, causing degeneration due to the lack of neurotrophic factors. For this reason, both 5-HT and TH markers are useful for assessing the effects that these treatments have on axonal regeneration. These fibers modulate in some way locomotor network (43–45).

The results of our study show that the microenvironment created in rats treated with INDP alone is associated with a significant increase in the number of 5-HT+ and TH+ fibers located in the caudal segment of the SC and so improving locomotor activity. Differently, in the group receiving the combination therapy there was no difference in the number of these fibers. It is important to mention that the minimal motor recovery observed could be due to other tracts such as corticospinal tracts, which are not evaluated in this study. It is known that an injury inflicted by contusion originates a stronger reaction and, thereby, a greater tissue destruction compared to an injury inflicted by transection (46). This could provide more opportunities in axonal regeneration for the latter and less for the first one. In addition, the resulting different microenvironment could likely affect the capacity to induce axonal regeneration-specially for certain tracts- and probably supporting the regenerative process of other different fibers (47). This could partially explain the contrasting results observed -in axonal regeneration- between the present study and those previously reported in rats with SC transection (10). On the other hand, the lack of regeneration of 5-HT+ and TH+ fibers also contrasts with previous findings reported in the acute phase of injury after SC contusion. The acute phase of injury is characterized by an intense inflammatory response and a great release of neural constituents (48). These factors can better activate the protective and restorative effects of the combination therapy to induce the regeneration of the tracts mentioned above. In contrast, the chronic phase is a period of generalized stability in which many of the elements that were activated as protective means or promoters of the restoration observed during the acute phase of the injury are missing (49, 50). Although in our model the SR inflicted a slight injury, it was not enough to support the beneficial effects of the combination therapy and, thereby, the regeneration of the evaluated tracts.

We also evaluated neurogenesis and observed that only the group treated with INDP alone shows a significant increase in the number of early formed neurons. The results found in this study suggest that the combination strategy did not act in synergy with INDP and more than improving, it inhibits its beneficial effects after a chronic SC contusion. This unexpected result, is the opposite of what has been observed in models of chronic SC transection where a more favorable result was obtained (10). These contrasting results demonstrate again that the microenvironment generated after a SC transection or contusion are different and therapeutic strategies should be individualized.

Some limitations of the present study, in the failure of the combination therapy, might be the ideal timing. Previous studies in our group have shown that INDP requires T cells activation and their interaction with other immune cells (51). In addition, when anti-inflammatory or immunosuppressive treatments, such as Methylprednisolone or Cyclosporin A are administrated at the same time with A91 peptide, INDP effect is not observed. The combination therapy used in this investigation uses simultaneously INDP and MSCs. Therefore, MSCs could interfere with INDP action due to their anti-inflammatory effects, which would show a similar result as the ones with methylprednisolone or Cyclosporin A. This is a topic that should be addressed in further studies. Future investigations must be carried out and focused in the assessment of whether the INDP could have a better effect when applied a few days before the MSCs transplant instead of being used simultaneously. Additionally, some complementary evaluations as electrophysiological studies should be included in order to improve the functional analysis.

## Conclusion

The findings observed in this study, suggest that the combination therapy (SR + INDP + FGM-MSCs) modifies the non-permissive microenvironment post spinal cord injury, but it is not capable of inducing an appropriate axonal regeneration or neurogenesis when compared to the treatment with INDP alone. Therefore, indicating that the best therapy in a chronic SC contusion rat model is purely with INDP.

## Data Availability Statement

All datasets generated for this study are included in the article/supplementary material.

## Ethics Statement

The animal study was reviewed and approved by Mexican Official Norm on Principles of Laboratory Animal Care (NOM 062-ZOO-1999). In addition, the Animal Bioethics and Welfare Committee approved all animal procedures (ID: 178544; CSNBTBIBAJ 090812960).

## Author Contributions

RR-B contributed to the concept, design of experiments, as well, substantially contributed to the acquisition, analysis, interpretation of data, and drafting of the manuscript. AF-R contributed to acquisition and interpretation of data, surgical procedures and postoperative care of the experimental animals. VB-A contributed to the acquisition, analysis, interpretation of data. EG contributed to the surgical procedures. KS-Z was contributed to surgical procedures and postoperative care of the experimental animals. DI-A was involved to the acquisition and contributed in the drafting of the manuscript and postoperative care of the experimental animals. MG-L was involved to the acquisition and postoperative care of the experimental animals. JJ-VW was substantially involved in the drafting of the manuscript and postoperative care of the experimental animals. AI contributed to the conception and design of this project, general supervision of the research group and gave final approval of this manuscript. All authors read and approved the final manuscript.

### Conflict of Interest

The authors declare that the research was conducted in the absence of any commercial or financial relationships that could be construed as a potential conflict of interest.

## Abbreviations

CICSA: Centro de Investigación en Ciencias de la Salud
CMN Siglo XXI: Centro Médico Nacional Siglo XXI
INDP: Immunization with neural derived peptides
SR: Scar removal
MSCs: Mesenchymal stem cells
SCI: Spinal cord injury
FGM: Fibrin glue matrix
FGM-MSCs: Fibrin glue matrix with MSCs
CONACyT: National Council of Science and Technology of Mexico
SC: Spinal cord
CNS: Central Nervous System
PA: Protective autoimmunity
MBP: Myelin basic protein
5-HT: Serotonin
BDNF: Brain derived neurotrophic factor
GAP-43: Growth associated protein-43
IL-4: Interleukin 4
IL-10: Interleukin 10
TNFα: Tumor necrosis factor alpha
INFγ: Interferon gamma
TGFβ: Transforming growth factor beta
IGF-1: Insulin-like growth factor 1
TH: Tyrosine hydroxylase
NOM 062-ZOO-1999: Mexican Official Norm on Principles of Laboratory Animal Care
DMEM: Dulbecco's Modified Eagle
FBS: Fetal bovine serum
DPY: α,α′- dipyridyl
PBS: Phosphate buffered saline
CFA: Complete Freund's adjuvant
BBB: Basso, Beattie & Bresnahan
ELISA: Enzyme-linked immunosorbent assay
BrdU: 5-bromo-2′-deoxyuridine
Dcx: Doublecortin
NPCs: Neural progenitor cells
PBT: PBS-Triton
DAPI: 4′,6-diamidino-2-phenylindole
SD: Standard deviation
BBB test: Bonferroni's *post hoc* test
ERK: Extracellular Signal-regulated Kinase
STAT 3: Signal transducer and activator of transcription 3
Bcl-2: B-cell lymphoma 2
Bcl-xL: B-cell lymphoma extra-large
CD4+: Cluster of differentiation 4
AKT: Protein kinase B
MAPK: Mitogen-activated protein kinase
INFγ: Interferon gamma
TGFβ: Transforming growth factor beta
ANOVA: Analysis of variance.
